# Characterization of Endothelium-Dependent Relaxation in the Saphenous Artery and Its Caudal Branches in Young and Old Adult Sprague Dawley Rats

**DOI:** 10.3390/biom12070889

**Published:** 2022-06-25

**Authors:** Andrea N. Wang, Graham M. Fraser, John J. McGuire

**Affiliations:** 1 Department of Medical Biophysics, Schulich School of Medicine & Dentistry, Western University, London, ON N6A 5C1, Canada; awang293@uwo.ca; 2 Division of BioMedical Sciences, Faculty of Medicine, Memorial University, St. John’s, NL A1B 3V6, Canada; graham.fraser@med.mun.ca; 3 Department of Physiology and Pharmacology, Schulich School of Medicine & Dentistry, Western University, London, ON N6A 5C1, Canada

**Keywords:** endothelium, nitric oxide, proteinase-activated receptor 2, ageing, cardiovascular system

## Abstract

Ageing is associated with reduced endothelium-derived nitric oxide (NO) production in the femoral artery of Sprague Dawley (SD) rats. In the current study, we examined endothelium-dependent relaxation (EDR) in the saphenous artery and its caudal branches. We used acetylcholine and the Proteinase-Activated receptor-2 (PAR2)-specific agonist (2fLIGRLO) with nitroarginine methylester (L-NAME) to assess EDR in two groups of male SD rats (age in weeks: young, 10–12; old, 27–29). Acetylcholine and 2fLIGRLO were potent NO-dependent relaxant agents in all arteries. For all arteries, EDR by acetylcholine decreased significantly in old compared to young SD rats. Interestingly, PAR2-induced EDR of proximal saphenous artery segments and caudal branches decreased significantly in old compared to young, but did not differ for the in-between middle and distal ends of the saphenous artery. L-NAME treatment increased subsequent contractions of proximal and middle segments of saphenous arteries by phenylephrine and U46619 in young, but not in old, SD rats. We conclude the SD saphenous artery and caudal branches exhibit regional characteristics that differ in response to specific EDR agonists, endothelial NO synthase inhibitor, and changes to endothelium function with increased age, which are, in part, attributed to decreased sensitivity of vascular smooth muscle to the gaseous transmitter NO.

## 1. Introduction

In healthy persons, dynamic regulation of blood perfusion of the limbs and skeletal muscles enables mobility across a range of aerobic intensities. Decreases in oxygen perfusion are associated with a decline in mobility with age [1], which may lead to disability and fragility [2]. The normal mechanisms that provide skeletal muscles with higher levels of perfusion, e.g., during active use, to meet an increased demand of oxygen [3,4], are impaired in ageing [5] and similarly by diseases, such as type 2 diabetes and peripheral artery diseases [6,7]. A loss or decrease in the normal functions of the vascular endothelium is a key mechanism underlying vascular changes associated with age and diseases [8,9,10].

The endothelium produces nitric oxide and other gaseous transmitters that are mediators of endothelium function [11,12]. In humans, impaired endothelium-dependent relaxation (EDR) mechanisms have been implicated in studies showing that vasodilation of forearm and leg arteries, and microvasculature are reduced with age [13,14,15,16,17]. Similarly, studies in animal models of cardiovascular disease show reduced efficacy for EDR with ageing [18,19,20]. Decreased NO synthesis and bioavailability and decreased NO sensitivity of downstream targets in vascular smooth muscle are signaling mechanisms associated with impaired endothelium function with age [19,21,22,23,24,25]. However, EDR mechanisms themselves vary according to the blood vessel type, and, thus, understanding changes to endothelium function necessitates specific knowledge of the relevant vasculature.

The majority of blood flow to the hind limb and foot in the Sprague Dawley (SD) rat passes through the femoral artery [26,27]. Normal ageing is associated with reduced endothelium-derived NO in this conduit-type blood vessel in rats [28,29,30,31] and mice [32,33]. Conversely, evidence for preserved vasodilation in the femoral artery with age has also been reported in studies of rats [19,26], dogs [34,35], and humans [36]. However, in vessels further downstream of the femoral artery, specifically in the saphenous artery and its branches, endothelial function has yet to be elucidated. In rodents, the femoral artery bifurcates into the popliteal and saphenous artery [37]. The saphenous artery feeds the various tissues within the foot and, also, has branches that supply the medial thigh muscles [27]. Endothelium-dependent relaxation mechanisms have not been explored in the most distal hind limb arterial vasculature of healthy ageing rats [28,29]. Similarly, the vasoreactivity in the saphenous artery and branches have not been explored in detail [22]. Identification of EDR mechanisms provides insight that may be used to develop therapeutic strategies to treat diseases and pathology resulting from endothelium dysfunction.

Protease-activated receptor-2 (PAR2) is a seven transmembrane domain class-A G protein-coupled receptor (GPCR) expressed constitutively on the vascular endothelium of blood vessels, various epithelial tissues, and immune cells [38]. PAR2 is activated by trypsin, trypsin-like serine proteases and several other proteases, some of which are released by tissue injury and inflammatory cells, through a tethered-ligand generated by cleavage of its extracellular amino-terminus [38]. The synthetic PAR2-activating peptide 2-furoyl-LIGRLO (2fLIGRLO) is a pharmacological ligand, which is based on the tethered-ligand sequence for PAR2 that, upon binding to the receptor, stimulates signaling pathways and endothelium-mediated responses, as trypsin does without generating the tethered ligand [39]. Studies show PAR2-mediated endothelium-dependent relaxations are often preserved when endothelial cell responses elicited by other GPCR agonists, such as acetylcholine (ACh) and bradykinin, are reduced in animal models of disease, including hypertension, stroke, diabetes, obesity, and metabolic syndrome [40,41,42]. Further time-course studies using a rat model of metabolic syndrome show the preserved PAR2 mediated EDR eventually deteriorating with the progression of disease severity, which is associated with the age of the animals and varies by vasculature [43]. Additional studies of age and time-dependent changes to PAR2-mediated responses of the vasculature may provide insight into mechanisms and the potential for targeting this receptor for therapeutic treatment of endothelium dysfunction.

In this study we aimed to develop a model to characterize EDR through a muscarinic, and PAR2-specific agonist in the saphenous artery and its caudal branches in young and old adult male SD rats. Using this model, we examined the differences between groups for the baseline and agonist-induced NO production in different segments of the saphenous artery and its branches. The implication of our findings suggests that further studies of diseases and strategies to develop therapeutics need to account for these blood-vessel-specific characteristics.

## 2. Materials and Methods

### 2.1. Animals

All procedures involving animals were approved by the Institutional Animal Care Committee at Western University in accordance with guidelines and principles by the Canadian Council on Animal Care. Male Sprague Dawley rats were purchased from Charles River Laboratory (PQ). Rats were group housed (2–5 per cage) and provided a standard laboratory rodent diet (Prolab 3000) and water ad libitum. Housing rooms were set for 12 h light (7 am to 7 pm) and 12 h dark cycle (7 pm to 7 am) with an environmentally controlled room temperature (20–24 °C) in the Animal Care Health Sciences Facility at Western University. Animals were assigned to two groups: young (*n* = 31, 10–12 weeks of age) and old (*n* = 6, 27–29 weeks of age). On the morning of an experiment, the rat was transported to the laboratory, weighed, and then euthanized by cervical dislocation under isoflurane anesthesia.

### 2.2. Drugs and Chemicals

All drugs and reagents were purchased from Sigma Aldrich Canada (Oakville, ON, Canada) unless stated otherwise. 2-furoyl-Leu-Ile-Gly-Arg-Leu-Orn-NH_2_ (2fLIGRLO) was purchased from GenScript (Piscataway, NJ, USA) and a stock solution (5 mM) prepared using 25 mM HEPES, pH 7.4. Acetylcholine chloride (ACh), phenylephrine hydrochloride (PE), and sodium nitroprusside (SNP) were purchased from BioBasic Canada (Markham, ON, Canada). Stock solutions of ACh, PE, SNP, and U46619 (Cayman Chemical Company, Ann Arbor, MI, USA) were prepared in ultra-purified molecular biology-grade water.

### 2.3. Isolation of Hind Limb Arteries

**Figure 1:**
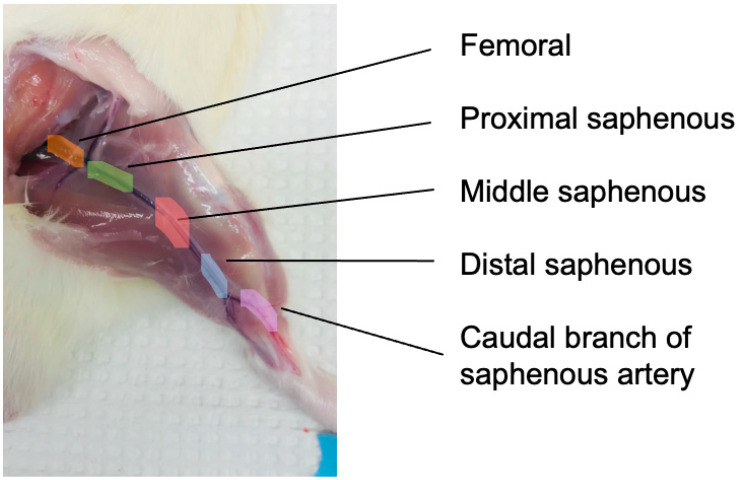
Anatomical representation of hind limb arteries in male Sprague Dawley rat. The femoral artery (orange) is identified as the artery prior to bifurcation into popliteal and epigastric arteries. The saphenous arteries were separated into proximal (green), middle (red), and distal (blue) segments based on distance to the femoral artery. The caudal branch (purple) is one of the bifurcations of the saphenous artery.

Specific anatomical reference points for the methods are depicted in Figure 1 below.

The skeletal muscle of the hind limb containing the blood vessels of interest was dissected in situ and immediately placed into ice-cooled Krebs-HEPES buffer (pH 7.4) comprising 114 mM NaCl, 4.7 mM KCl, 0.8 mM KH_2_PO4, 1.2 mM MgCl_2_ 6H_2_O, 2.5 mM, CaCl_2_ 2H_2_O, 11.0 mM D-Glucose, 20 mM NaHCO_3_, and 5 mM HEPES hemisodium salt. The femoral, saphenous, and caudal branch of saphenous artery were isolated from the hind limb skeletal muscle and cleaned of surrounding fat, the femoral and saphenous nerves and veins. Each blood vessel was cut into 1–2 mm length rings and the saphenous artery was subdivided according to the distance from femoral artery into proximal, middle, and distal segments.

### 2.4. Relaxation Bioassays with Hind Limb Arteries

These methods follow those previously described in [44] with modifications. We inserted two silver-plated tungsten wires (40 µm diameters) through the lumen of each ring and mounted rings between two jaws of the myograph while suspended in chambers (DMT 620M) containing Krebs-HEPES buffer (pH 7.4) bubbled with 95% O_2_/5% CO_2_ at 37 °C throughout the duration of the experiment. Data measurements were collected and recorded simultaneously from 12 vessels of each rat. The initial resting tension of blood vessels was determined for each artery segment such that the vessels were stretched to a target (IC_1_) internal circumference equal to 90% (IC_1_/IC_100_ = 0.9) of their internal circumference (IC_100_) at an effective target pressure of 13.3 kPa (100 mmHg); this was modified from [45].

Blood vessels were held at resting tension to equilibrate for 30 min prior to the addition of any drugs. Isometric tensions of the blood vessels were recorded continuously while drugs were added directly to the baths. A dose response curve of KCl (30, 60, 90 mM) was used to test viability of preparations. Vessels were excluded when contractions by 90 mM KCl were less than 2 mN. Cumulative concentration–response curves (CRCs) were constructed for phenylephrine (PE, 1 nM–10 µM), U46619 (1 nM–1 µM), ACh (1nM–10µM), and 2fLIGRLO (1 nM–3 µM) in untreated (controls), and NO synthase (NOS) inhibitor-treated arteries (L-NAME, 100 µM for 10 min prior to precontraction of the arterial rings) [19,26,44]. For relaxation assays, we used U46619 to produce submaximal contractions (<90% of maximum) in all vessels. Specifically, we used the following concentrations of U46619: femoral artery, 0.5 µM; proximal and middle saphenous arteries, 0.1 µM; and distal and caudal branches of saphenous artery, 0.2 µM. Following each CRC that was conducted, the chambers were rinsed >5 times with Krebs-HEPES buffer for a 30 min washout period. After the last concentration of ACh and 2fLIGRLO, we measured the relaxations by a single dose of SNP (1 µM), a maximally effective concentration.

In this study, the design and execution of drug treatment protocols were constrained by the requirement to develop and optimize methods for studying the saphenous arteries and caudal branches. All treatments could not be carried out in the same vessel segments. Phenylephrine CRCs were determined in separate segments in order to avoid desensitization in response to repeated tests which we had observed in a small set of the first series of experiments. U46619 CRCs with control and L-NAME treatments were carried out on the same and separate vessel segments. During the initial experiments to optimize isolation procedures for viable vessels from younger animals, both U46619 and PE were tested in the same segments and we randomized the order. ACh and 2fLIGRLO CRCs were determined in separate and the same vessel segments. In these cases, ACh and 2fLIGRLO responses were determined prior to PE and U46619 CRCs.

### 2.5. Data Analysis

Data are reported as the mean ± standard error of mean (SEM) unless otherwise indicated; *n* = number of independent experiments (rats), each with two to three replicates for each type of vessel. On the graphs, the lines are the concentration–response curves determined by nonlinear regression curve fitting using best-fit sigmoidal dose response equations calculated with GraphPad Prism version 9.4.0 for Windows (GraphPad Software, San Diego, CA, USA). For each drug, the maximum effects (E_max_) for contraction or % relaxation were calculated directly from the responses of each arterial ring and averaged for each rat. Drug potency (-logEC_50_ where EC_50_ is the effective drug concentration (M) producing 50% of E_max_) and Hill slope (n_H_) were determined from the best-fit curves for each group. E_max_ for U46619 and PE are expressed as a percentage of the contractions elicited by 90 mM KCl in the same artery.

(1) $$\mathrm{Contraction}\;\left(\%\right)=\frac{\mathrm{tension}\;\mathrm{with}\;U46619\;\mathrm{or}\;\mathrm{PE}\;(\mathrm{mN})\;-\;\mathrm{resting}\;\mathrm{tension}\;\left(\mathrm{mN}\right)}{\mathrm{tension}\;\mathrm{with}\;90\;\mathrm{mM}\;\mathrm{KCl}\;(\mathrm{mN})\;-\;\mathrm{resting}\;\mathrm{tension}\;\left(\mathrm{mN}\right)}$$

E_max_ for relaxation by ACh, 2fLIGRLO, and SNP is calculated from the reversal of the precontraction tone where 100% relaxation is a complete reversal of agonist-induced contraction.

(2) $$\mathrm{Relaxation}\;\left(\%\right)=\frac{\mathrm{tension}\;(\mathrm{mN})\;\mathrm{with}\;U46619\;-\;\mathrm{tension}\;\mathrm{with}\;\mathrm{ACh},\;2\mathrm{fLIGRLO},\;\mathrm{SNP}\;\left(\mathrm{mN}\right)}{\mathrm{tension}\;(\mathrm{mN})\;\mathrm{with}\;U46619\;-\;\mathrm{resting}\;\mathrm{tension}\;(\mathrm{mN})}$$

Unpaired Student’s *t*-test was used to compare two groups. One- or two-way analysis of variance (ANOVA) was used to compare more than two groups and followed by multiple comparison testing using Bonferroni post-hoc tests. *p* < 0.05 was considered significant.

## 3. Results

### 3.1. Physical Characteristics of Young and Old SDs and Their Hind Limb Arterial Ring Segments

Body masses of SDs in the old group were 1.8-times higher than in the young group (in grams, 663 ± 104 vs. 376 ± 80). Physical characteristics of the artery segments for tension studies are shown in Table 1.

Contractions of saphenous artery distal segments by K^+^ (90 mM) were 1.2-times higher in old vs. young SDs (*p* < 0.05). However, these contractions in the proximal and middle segments and caudal branches did not differ between old and young SD.

### 3.2. Baseline NOS Inhibition in Young SDs Produces Selective Increases in PE-Mediated Contractions by Artery Type

**Figure 2:**
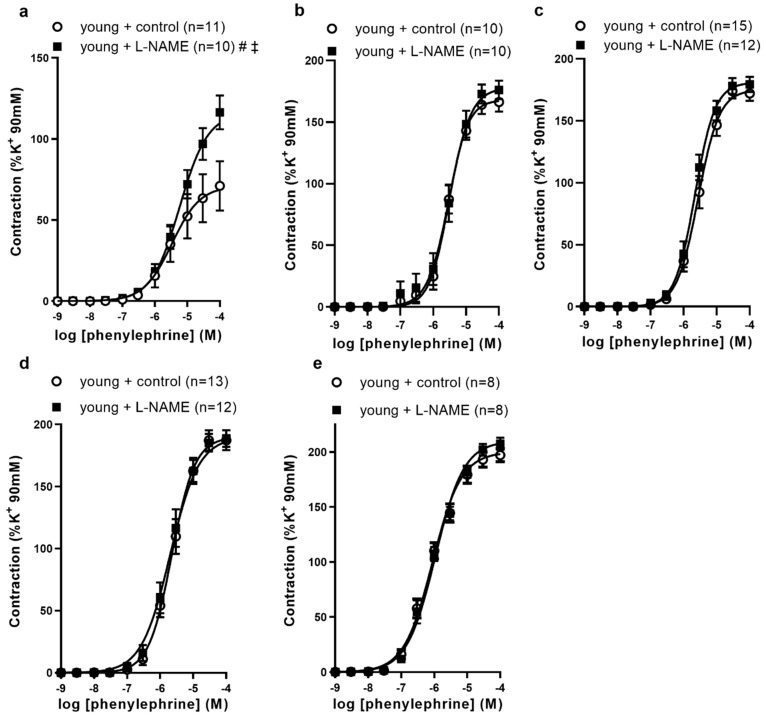
The effects of NO synthase inhibitor L-NAME on phenylephrine-induced contractions of (**a**) femoral, (**b**) proximal, (**c**) middle, (**d**) distal saphenous artery segments, and (**e**) caudal branches from young Sprague Dawley rats. The results are expressed as mean ± S.E.M of (*n*) rats. Lines represent best-fit four parameter sigmoidal dose response curves. # *p* < 0.05, vs. E_max_ control; ‡ *p* < 0.05, vs. −logEC_50_ control by Student’s *t*-test.

First, we examined whether NOS inhibitor effects on the contraction by phenylephrine (PE) differed by the artery segment (shown in Figure 2). At baseline tension in young animals, femoral (shown in Figure 2a) artery contractions by PE were 1.4-times higher than in controls (*p* < 0.05, E_max_). Phenylephrine contractions of the saphenous artery segments and caudal branches did not differ between control and L-NAME treatment (shown in Figure 2c–e).

These data suggested basal NO opposing adrenoceptor agonist vasoconstrictor activity is diminished moving downstream from the femoral artery (shown in Table 2).

### 3.3. Baseline NOS Inhibition Produces Selective Increases in U46619-Mediated Contractions by Artery Type, Which Diminishes in Old Sprague Dawley Rats

**Figure 3:**
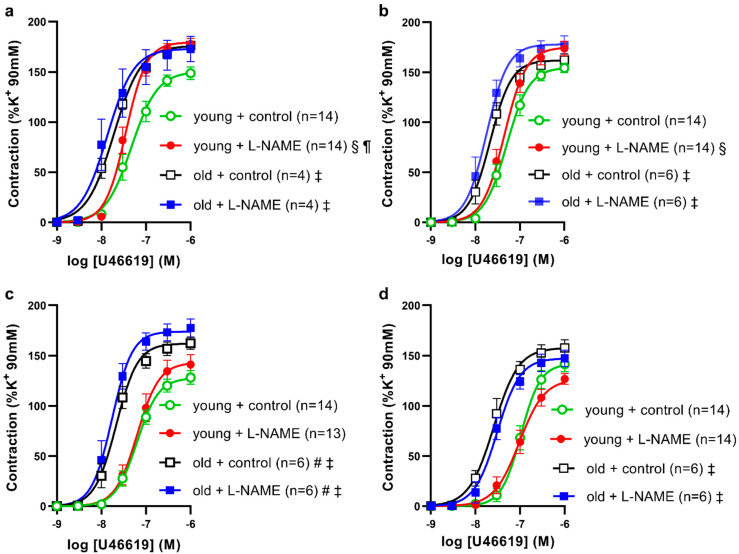
The effects of NO synthase inhibitor L-NAME on U46618-induced contractions of (**a**) proximal, (**b**) middle, (**c**) distal saphenous artery segments, and (**d**) caudal branches from (circle symbols) young and (square symbols) old Sprague Dawley rats. The results are expressed as mean ± S.E.M of (*n*) rats. Differences in best-fit curve parameters were compared by 2-way ANOVA, followed by Bonferroni post-hoc test. Lines represent best-fit four parameter sigmoidal dose response curves. # *p* < 0.05, vs. E_max_ young + control; ‡ *p* < 0.05, vs. −log EC_50_ young + control; § *p* < 0.05, E_max_ L-NAME vs. control; ¶ *p* < 0.05, −log EC_50_ L-NAME vs. control.

Next, we examined whether NOS inhibitor effects on contractions varied with U46619, a thromboxane receptor agonist, and assessed differences between the young and old SDs (shown in Figure 3). In young SDs, the contractions of proximal saphenous (shown in Figure 3a) and middle saphenous (shown in Figure 3b) arteries by U46619 were 1.2-, and 1.1-times increased by L-NAME treatment, and sensitivity to U46619 increased 1.2-times in L-NAME vs. control-treated proximal segments. Contractions of the distal saphenous (shown in Figure 3c) and caudal branches (shown in Figure 3d) by U46619 were not significantly different between control and L-NAME treatments in young SDs. These data are consistent with the findings with PE showing differences in basal NOS capacity to oppose agonist-induced vasoconstriction, diminished with increasing distance from the femoral artery.

In old SDs, the sensitivities of artery segments to U46619 were increased three-times in the proximal, middle, and distal saphenous artery segments and four-times in caudal branches, compared to young SDs (shown in Figure 3a–d). Contractions of proximal and distal segments at 1 µM U46619 in the old (control) group were increased 1.2- and 1.3-times, respectively, compared to young (control) group. Maximal contractions of middle and caudal branches by U46619 did not differ significantly between young and old SDs. In all arteries of old SDs, the U46619 CRC best-fit parameters did not differ significantly between control and L-NAME treatments. Baseline tensions of arteries did not differ between L-NAME and control treatments nor between old and young groups (*p* > 0.05, control vs. L-NAME, Student’s paired *t*-test (femoral arteries) and 2-way ANOVA with Bonferroni (all other groups)). We interpret these data (shown in Table 3) as evidence for diminished NOS inhibition of vasoconstrictor agonist-induced tone in the proximal and middle saphenous artery segments in the old SDs.

### 3.4. Agonist and Vessel-Type Selective Relaxation Effects in Hind Limb Arteries of Young and Old SDs

**Figure 4:**
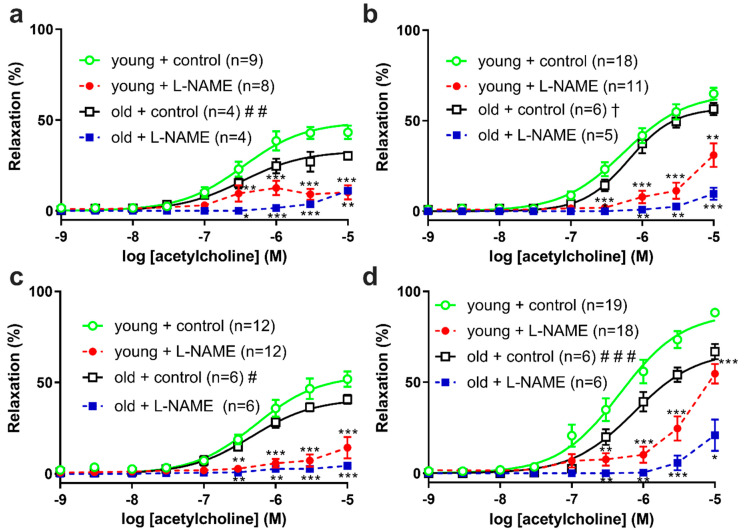
Acetylcholine-induced relaxations of (**a**) proximal, (**b**) middle, (**c**) distal saphenous artery segments, and (**d**) caudal branches from (circle symbols) young and (square symbols) old Sprague Dawley rats. The results are expressed as mean ± S.E.M of (*n*) rats. Lines represent best-fit four parameter sigmoidal dose response curves for controls. Differences in best-fit curve parameters for controls were compared by Student’s *t*-test. # *p* < 0.05, ## *p* < 0.01, ### *p* < 0.001, vs. E_max_ young; † *p* < 0.05, vs. nH young. Differences in relaxations in control vs. L-NAME treatment were compared by 2-way ANOVA with Bonferroni post hoc. * *p* < 0.05, ** *p* < 0.01, *** *p* < 0.001, vs. age-matched control.

Different agonists are known to produce different EDR mechanisms that vary according to the blood vessel type and disease model. In hind limb arteries, we examined relaxations of saphenous arteries and their caudal branches by ACh in young vs. old SDs (shown in Figure 4). Compared to young SDs, the proximal, middle, and distal segments’ maximum relaxations by ACh were 0.63-, 0.77-, and 0.81-times decreased in old SDs (shown in Figure 4a–c). The caudal branches’ maximum relaxations by ACh were 0.75-times decreased in old compared to young SDs (shown in Figure 4d). Further, in caudal branches, the sensitivity to ACh was 1.6-times decreased in old vs. young SDs. Together, these data were interpreted as evidence for reduced EDR in old SDs (shown in Table 4).

In the young SD group, acetylcholine-induced relaxations of U46619 (0.5 µM)-contracted femoral arteries were 88% inhibited by L-NAME compared to control treatment (*p* < 0.0001, Student’s paired *t*-test, control vs. L-NAME: *n* = 9, 9; E_max_ 65 ± 5 % vs. 8 ± 3 %; mean difference control − L-NAME, 57 ± 5%). In femoral artery segments (controls), the −logEC_50_ and n_H_ values were 6.72 ± 0.04 and 1.7 ± 0.2, respectively. Together with the data from saphenous arteries and end branches in young animals (shown in Table 4), E_max_ values for ACh CRCs were negatively correlated with larger internal diameters of arteries, indicating internal diameters accounted for <10% of the total variation (r = −0.31, *p* < 0.01, *n* = 72 paired data).

**Figure 5:**
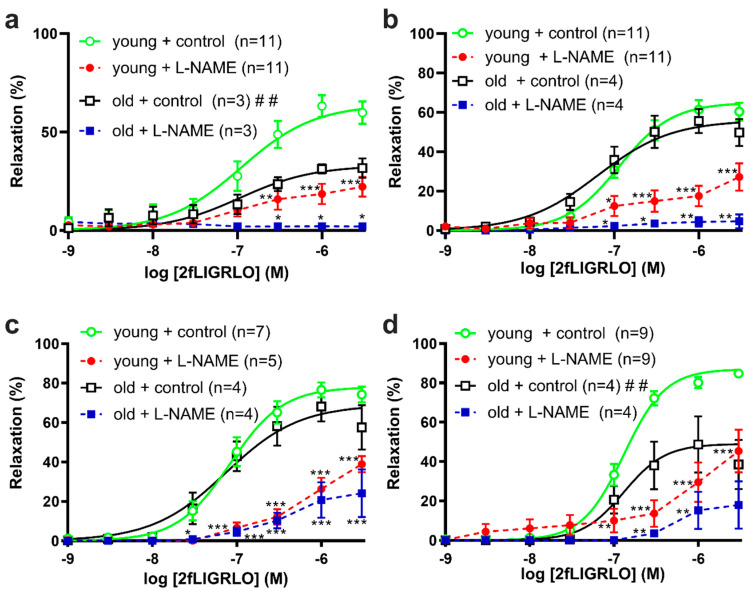
PAR2-specific agonist-induced relaxations of (**a**) proximal, (**b**) middle, (**c**) distal saphenous artery segments, and (**d**) caudal branches from (circle symbols) young and (square symbols) old Sprague Dawley rats. The results are expressed as mean ± S.E.M of (*n*) rats. Solid lines represent best-fit four parameter sigmoidal dose response curves for controls. Differences in best-fit curve parameters for controls were compared by Student’s *t*-test. ## *p* < 0.01, vs. E_max_ young control. Differences in relaxations of control vs. L-NAME treatment were compared by 2way ANOVA with Bonferroni post hoc. * *p* < 0.05, ** *p* < 0.01, *** *p* < 0.001, vs. age-matched control.

Previous studies have implicated different agonists as producing different mechanisms of relaxation. Next, we examined hind limb artery relaxations by the PAR2-specific agonist 2fLIGRLO (shown in Figure 5). The proximal saphenous artery and caudal branches’ maximal relaxations by 2fLIGRLO were 0.59- and 0.62-times decreased in old compared to young SDs (shown in Figure 5a,d). For middle and distal saphenous artery segments, the 2fLIGRLO potencies were 1.6- and 1.4-times decreased in old compared to young SDs (shown in Figure 5b,c and Table 4).

We used L-NAME to assess the NOS-dependent mediators of EDR in young and old SDs. In the segments of saphenous arteries and caudal branches from both age groups, L-NAME treatments (as shown by dashed lines in Figure 4 and Figure 5) inhibited relaxations by ACh and 2fLIGRLO. In L-NAME-treated caudal branches from young SDs (shown by dashed lines in Figure 4d and Figure 5d), the residual relaxations by ACh and 2fLIGRLO were more apparent than in other arteries (shown by dashed lines in Figure 4a–c and Figure 5a–c).

**Figure 6:**
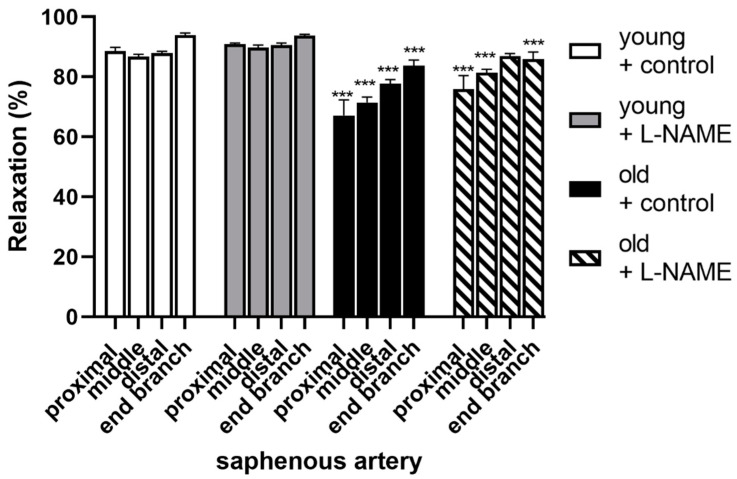
Nitroprusside-induced relaxations of proximal, middle, distal saphenous artery segments, and caudal (end) branches from (white and grey bars) young and (black and striped bars) old Sprague Dawley rats. Arteries treated as either controls (white and black bars) or with L-NAME (grey and striped bars). Relaxation % is the reversal of active tension by addition of 1 µM nitroprusside in U46619-precontracted arteries. The results are expressed as mean ± S.E.M (young, *n* = 3–18; old, *n* = 3–6). Differences in relaxations of young vs. old were compared by 2way ANOVA with Bonferroni post hoc. *** *p* < 0.001, vs. treatment-matched artery in young group.

We also assessed vascular smooth muscle relaxations by nitroprusside (SNP), which causes relaxation via a mechanism independent of the endothelium. In proximal, middle, distal saphenous arteries, and caudal branches, the relaxations by SNP (1 µM) were 0.75-, 0.82-, 0.88-, and 0.89-times decreased, respectively, in old vs. young SDs (shown in Figure 6). We considered that enhanced contractility of vascular smooth muscle in L-NAME-treated arteries may have produced a functional antagonism of the relaxations by EDR agonists and NO; however, the relaxations by SNP did not differ in the presence of L-NAME (shown in Figure 6).

## 4. Discussion

The main finding from this study of EDR in the saphenous arteries and their caudal branches is that endothelium NO synthase activity opposing vasoconstrictor agonist-induced tone and endothelium GPCR agonist-stimulated NO release decreased with the increased age of SDs. This study is the first to characterize different segments of the saphenous arteries and their caudal branches in SDs. We found evidence of saphenous artery region-selective responses to different agonists and NO synthase inhibitor. Our data implicate the gaseous transmitter NO as being the primary mediator of vasodilation in the saphenous arteries of SD, albeit the presence of non-NO mediators is evident towards the distal segment and end branches. Together, these findings indicate surprising regional differences for both agonist-induced and basal NO in the saphenous arterial blood supply to the rat hind limb.

Our study of the saphenous artery adds to the evidence that changes in endothelial function occur across all levels of vasculature in the rat. In previous studies, for example, evidence is provided for reduced endothelial-dependent vasodilation by ACh or bradykinin with rat aortas [19,26], femoral [28,29], mesenteric arteries [46,47], basilar arteries [48], and feed arteries and arterioles of the soleus muscle [20,49,50]. Interestingly, one study reported that ACh-induced vasodilation of the saphenous artery did not differ between 12, 34, and 64 weeks of age in mice [22]. We segmented the saphenous artery into regions, which is a notable difference in methods. The mechanisms underlying the regional selective endothelium responses within the rat saphenous artery are as yet undetermined. Heterogeneous vasodilator responses in iliac, femoral, and gastrocnemius feed arteries of aging rats are evidence that pathways associated with vasodilation vary with the internal lumen diameters [26]. The data from resting (passive) tension normalization procedures in our study imply a relatively constant lumen diameter for the rat saphenous artery from its origin at the femoral artery to bifurcation at the caudal branches. Initially, we expected the pharmacological responses would not differ between the saphenous artery segments. However, we observed some subtle morphological differences within these regions, including the presence of small daughter branches. Endothelium exposure to different wall shear stress can lead to different endothelial cell phenotypes, i.e., more prone to dysfunction and atherosclerosis. We speculate the physical differences within segments of the saphenous artery could produce regional differences in apparent wall shear stress that the endothelium is exposed to under normal blood flow and, thus, produce some heterogeneity of endothelium function, characterized here as modest changes in endothelium-derived NO. In the caudal arteries bifurcating from the saphenous artery, a significant non-NO synthase-dependent mechanism was involved with ACh and 2fLIGRLO-induced relaxations. The age-dependent changes to EDR did not differ across the saphenous artery and caudal branches, which suggests the inter-regional differences in EDR were overall constant with time. In our previous work, we provided evidence that PAR2 ligand selective resistance to endothelium dysfunction was associated with differences in the intracellular calcium signals elicited by the ACh and PAR2 agonists, as shown by studies using fluorescence spinning disk confocal microscopy with isolated endothelial cells from mesenteric resistance arteries of normal and angiotensin-II-acquired hypertensive mice [51,52]. These molecular mechanisms warrant further study. It is interesting to speculate these branches of the saphenous artery mark a transition point in the predominance of NO vs. non-NO pathways that continues into the branching arterioles that feed the microvasculature of the lower leg and feet.

We found changes in ACh-induced EDR occurred with only a 16-week difference in ages of healthy SDs. Studies have reported similar findings after using younger and older rats, having 10- to 20-month age differences between groups [26,49,50,53]. The old SD animals in our study are similar in age to a ‘middle-age group’ used in a study that showed in-vivo administered eicosapentaenoic acid and docosahexaenoic acid increased agonist-induced EDR of femoral vein and arteries [28]. The decrease in PAR2-mediated EDR in some segments of saphenous arteries, and preserved function in others, suggest differing signal pathways for ACh and PAR2. Time-course studies of PAR2-mediated EDR implicate age as a factor in superior mesenteric arteries and aortas during the progressive development of chronic hypertension and metabolic syndrome, and show receptor-selective endothelial NOS-residue-targeted phosphorylation pathways for regulating NOS activity [43,54]. A study of forearm blood flow in healthy humans inferred the presence of agonist-specific endothelial vasodilator dysfunction by showing evidence that the forearm blood flow responses to ACh, but not bradykinin, and substance P, were ~30% lower in old than in young age groups [13]. In the current study, we used U46619 to contract tissues for the EDR assays that compared vessel segments from young to old SD rats. Phenylephrine, and U46619 are ligands that activate the α1-adrenoceptor and thromboxane prostanoid (TP) receptor, respectively. Both α1-adrenoceptor and TP are GPCR expressed on vascular smooth muscle cells and activate G_q/11_ and G_12/13_ signaling pathways. Relaxation responses elicited by vasodilators have been reported to differ between PE versus U46619-contracted tissues, as shown in a recent study on isolated human saphenous veins [55]. In our study, a decrease in the agonist EDR was not the only evidence of changes to vascular reactivity in the old compared to young SD. A difference in the NO release for countering increases in vascular tone in the femoral to saphenous and caudal arteries is evidenced by showing the NOS inhibitor treatment led to regional selective increases in contraction by vasoconstrictors. We also observed increases to vasoconstrictor agent sensitivity and decreases in direct acting NO, as shown with the increased contractions by U46619 and decreased relaxations by SNP, respectively, in old vs. young SDs. Interestingly, our current study results show saphenous arteries and their caudal branches have a higher sensitivity to TP agonists in old compared to young SDs. This effect of age was independent of changes to NO. Its mechanism and significance are undetermined; however, these vessels would be expected to be more sensitive to endothelium-derived TP-activating prostanoids, which previous studies showed are produced by the femoral and iliac arteries of Spontaneously Hypertensive Rats [56,57]. Together, these data show that the saphenous arteries and caudal branches can be used to detect significant differences in the vascular reactivity at a time-point approximating middle-age adult in the lifespan of the laboratory rat. Whether the changes are a normal physiological change with age or represent early detection of deteriorating endothelial regulation of vascular tone will require further investigation.

## 5. Conclusions

Nitric oxide is the predominant gaseous transmitter in the rat saphenous artery and its caudal branches. In the period from young to middle age in SDs, EDR decreased in a segment-selective manner along the saphenous artery and was accompanied by decreased vascular smooth muscle sensitivity to NO. These findings warrant consideration for the design of future studies to understand the mechanisms underlying endothelium dysfunction.

## Figures and Tables

**Table 1:** Blood vessel internal diameters and resting tension at baseline, and active tension induced by 90 mM extracellular K^+^ in hind limb arteries from young and old Sprague Dawley rats.

Artery	Group	*n*	Wall Length (mm)	Internal Diameter (µm)	Baseline Tension (mN/mm)	ΔK^+^ Contraction (mN/mm)
Femoral	young	15	3.81 ± 0.11	972 ± 46	2.87 ± 0.08	4.2 ± 0.3
Saphenous segment						
Proximal	young	31	3.69 ± 0.05	548 ± 11	1.45 ± 0.04	3.9 ± 0.2
	old	4	3.75 ± 0.05	609 ± 13	1.5 ± 0.2	5.2 ± 0.7
Middle	young	31	4.00 ± 0.05	578 ± 12	1.54 ± 0.06	4.5 ± 0.2
	old	6	4.00 ± 0.14	639 ± 23	1.55 ± 0.09	5.2 ± 0.3
Distal	young	31	3.93 ± 0.05	608 ± 13	1.62 ± 0.04	5.1 ± 0.2
	old	6	3.97± 0.12	690 ± 26 *	1.71 ± 0.08	6.3 ± 0.2 *
Caudal branch	young	25	3.78 ± 0.06	501 ± 10	1.19 ± 0.02	3.1 ± 0.1
	old	6	4.10 ± 0.17	588 ± 14 *	1.23 ± 0.07	3.2 ± 0.3

Femoral artery and saphenous artery segments and caudal branches were isolated from male Sprague Dawley rats comprising two groups: 31 young (10–12 weeks of age) and 6 old (27–29 weeks of age). Wall length = 2 × artery ring length. The lengths of arterial segments did not differ between groups. Internal diameter = IC_1_/π. Baseline (resting) tension (in mN per wall length) was recorded in artery rings mounted under isometric tension in wire-myograph using a normalization procedure with parameters: IC_1_ = 0.9 IC_100_ where IC_100_ is the estimated internal circumference of the arterial ring for an equilibrium target pressure equal to 13.3 kPa, based on length–tension relationships with each individual artery. ΔK^+^ contractions (in mN per wall length) = tension after addition of 90 mM KCl-resting tension. Values are mean ± S.E.M. of (*n*) rats with 1 to 5 replicates measured for each artery. Differences between groups for saphenous artery segments were compared by 2-way ANOVA with Bonferroni post-hoc test. * *p* < 0.05, old vs. young.

**Table 2:** The effect of pretreatment with L-NAME (100 µM) on phenylephrine concentration–response curves in hind limb arteries from young Sprague Dawley rats.

	Treatment	Femoral (11)	Proximal (10)	Middle (12)	Distal (11)	End Branch (8)
E_max_ (%)	Control	71 ± 15	168 ± 8	175 ± 6	190 ± 8	200 ± 6
	L-NAME	116 ± 10 *	178 ± 7	181 ± 6	190 ± 7	210 ± 5
n_H_	Control	1.00	1.5 ± 0.2	1.4 ± 0.1	1.2 ± 0.1	1.00
	L-NAME	1.00	1.3 ± 0.2	1.4 ± 0.1	1.2 ± 0.1	1.00
−log EC_50_ (M)	Control	5.47 ± 0.13	5.53 ± 0.04	5.56 ± 0.03	5.65 ± 0.04	6.05 ± 0.03
	L-NAME	5.24 ± 0.05 *	5.51 ± 0.05	5.65 ± 0.03	5.70 ± 0.04	5.97 ± 0.03

E_max_, maximum contraction response by PE, reported as % of active tension induced by 90 mM K^+^ in the same artery; −log EC_50_, negative logarithm of PE molar concentration producing 50% of E_max_; n_H_, Hill slope; PE, phenylephrine. EC_50_ and n_H_ were determined by nonlinear regression analyses of group data using sigmoidal dose–curve equations for best-fit curves. Values are mean ± S.E.M of (*n*) rats. Differences in best-fit curve parameters were compared by 2-way ANOVA with Bonferroni post-hoc test. * *p* < 0.05, vs. control.

**Table 3:** The effect of pretreatment with L-NAME (100 µM) on U46619 concentration–response curves in the saphenous artery segments and caudal branches from young and old Sprague Dawley rats.

	Treatment	Proximal		Middle		Distal		Caudal Branch	
		Young (14)	Old (4)	Young (14)	Old (6)	Young (14,13)	Old (6)	Young (14)	Old (6)
E_max_ (%)	Control	150 ± 6	176 ± 2	155 ± 5	162 ± 6	128 ± 7	162 ± 6 *	142 ± 7	158 ± 8
	L-NAME	180 ± 6 §	172 ± 13	175 ± 7§	178 ± 9	143 ± 9	178 ± 9 *	127 ± 6	147 ± 8
n_H_	Control	1.5 ± 0.2	1.4 ± 0.2	1.7 ± 0.2	1.8 ± 0.3	1.8 ± 0.2	1.8 ± 0.3	2.0 ± 0.3	1.6 ± 0.2
	L-NAME	1.9 ± 0.2	1.5 ± 0.2	1.8 ± 0.2	1.8 ± 0.3	1.8 ± 0.1	1.9 ± 0.4	1.5 ± 0.2	1.7 ± 0.3
−log EC_50_ (M)	Control	7.33 ± 0.04	7.72 ± 0.03 *	7.30 ± 0.04	7.67 ± 0.04 *	7.20 ± 0.04	7.67 ± 0.04 *	6.98 ± 0.04	7.59 ± 0.05 *
	L-NAME	7.45 ± 0.03 §	7.87 ± 0.05 *	7.35 ± 0.03	7.74 ± 0.05 *	7.20 ± 0.05	7.76 ± 0.05 *	7.02 ± 0.05	7.51 ± 0.04 *

E_max_, maximum contraction response by U46619, reported as % of active tension produced by 90 mM K^+^ in the same artery; −log EC_50_, negative logarithm of U46619 molar concentration producing 50% of E_max_; n_H_, Hill slope; U46619, thromboxane receptor agonist. EC_50_ and n_H_ were determined by nonlinear regression analyses of group data using sigmoidal dose–curve equations for best-fit curves. Values are mean ± S.E.M of (*n*) rats. Differences in best-fit curve parameters were compared by 2-way ANOVA with Bonferroni post-hoc test. * *p* < 0.05, vs. young + control; § *p* < 0.05, L-NAME vs. control.

**Table 4:** Concentration–response curve parameters for acetylcholine, 2fLIGRLO-induced relaxations of saphenous artery segments, and caudal branches from young and old Sprague Dawley rats.

Artery	Acetylcholine					2fLIGRLO			
		*n*	E_max_ (%)	−logEC_50_	n_H_	*n*	E_max_ (%)	−logEC_50_	n_H_
proximal	young	9	49 ± 3	6.46 ± 0.07	1.0	11	64 ± 5	7.0 ± 0.1	1.0
	old	3	32.7 ± 0.3 **	6.65 ± 0.05	1.0	3	33 ± 4 **	7.0 ± 0.1	1.0
middle	young	12	54 ± 4	6.27 ± 0.06	1.0	11	65 ± 4	6.94 ± 0.05	1.4 ± 0.2
	old	6	41 ± 2 *	6.33 ± 0.05	1.0	4	56 ± 6	7.19 ± 0.08	1.4 ± 0.1
distal	young	18	65 ± 3	6.26 ± 0.05	1.1 ± 0.1	7	78 ± 4	7.08 ± 0.05	1.4 ± 0.2
	old	6	57 ± 3	6.19 ± 0.02	1.4 ± 0.1 *	4	69 ± 8	7.14 ± 0.08	1.40 ± 0.3
caudal branch	young	19	88 ± 2	6.32 ± 0.05	1.0	9	87 ± 1	6.89 ± 0.03	1.8 ± 0.2
	old	6	67 ± 4 ***	6.13 ± 0.05	1.0	4	49 ± 14 **	6.88 ± 0.03	1.8 ± 0.2

E_max_, maximum relaxation response by acetylcholine (1 nM–10 µM), and PAR2 specific agonist 2fLIGRLO (1 nM–3 µM), reported as % of reversal of active tension in submaximal contracted U46619-treated arteries; −log EC_50_, negative logarithm of agonist molar concentration producing 50% of E_max_; n_H_, Hill slope; U46619, thromboxane receptor agonist. EC_50_ and n_H_ were determined by nonlinear regression analyses of group data using sigmoidal dose–curve equations for best-fit curves. Values are mean ± S.E.M of (*n*) rats. Differences in best-fit curve parameters were compared by unpaired Student’s *t*-test. * *p* < 0.05, ** *p* < 0.01, *** *p* < 0.001, vs. young.

## Data Availability

All data for this study are contained within the article and any additional data sharing will be considered by the corresponding author upon request.
